# Barriers and enablers to the delivery of psychological care in the management of patients with type 2 diabetes mellitus in China: a qualitative study using the theoretical domains framework

**DOI:** 10.1186/s12913-016-1358-x

**Published:** 2016-03-29

**Authors:** Anna Chapman, Hui Yang, Shane A Thomas, Kendall Searle, Colette Browning

**Affiliations:** School of Primary Health Care, Monash University, Building 1/270 Ferntree Gully Road, Notting Hill, Victoria, 3168 Australia; RDNS Institute, St Kilda, Victoria Australia; International Institute for Primary Health Care Research, Shenzhen City, Guangdong Province People’s Republic of China

**Keywords:** Type 2 diabetes mellitus, Psychological care, China, Theoretical domains framework, Guideline implementation, Evidence-based practice

## Abstract

**Background:**

China has the largest number of type 2 diabetes mellitus (T2DM) cases globally and individuals with T2DM have an increased risk of developing mental health disorders and functional problems. Despite guidelines recommending that psychological care be delivered in conjunction with standard T2DM care; psychological care is not routinely delivered in China. Community Health Centre (CHC) doctors play a key role in the management of patients with T2DM in China. Understanding the behavioural determinants of CHC doctors in the implementation of psychological care recommendations allows for the design of targeted and culturally appropriate interventions. As such, this study aimed to examine barriers and enablers to the delivery of psychological care to patients with T2DM from the perspective of CHC doctors in China.

**Methods:**

Two focus groups were conducted with 23 CHC doctors from Shenzhen, China. The discussion guide applied the Theoretical Domains Framework (TDF) that examines current practice and identifies key barriers and enablers perceived to influence practice. Focus groups were conducted with an interpreter, and were digitally recorded and transcribed. Two researchers independently coded transcripts into pre-defined themes using deductive thematic analysis.

**Results:**

Barriers and enablers perceived by doctors as being relevant to the delivery of psychological care for patients with T2DM were primarily categorised within eight TDF domains. Key barriers included: CHC doctors’ knowledge and skills; time constraints; and absence of financial incentives. Other barriers included: societal perception that treating psychological aspects of health is less important than physical health; lack of opinion leaders; doctors’ intentional disregard of psychological care; and doubts regarding the efficacy of psychological care. In contrast, perceived enablers included: training of CHC doctors in psychological skills; identification of afternoon/evening clinic times when recommendations could be implemented; introduction of financial incentives; and the creation of a professional role (e.g. diabetes educator), that could implement psychological care recommendations to patients with T2DM.

**Conclusions:**

The utilisation of the TDF allowed for the comprehensive understanding of barriers and enablers to the implementation of psychological care recommendations for patients with T2DM, and consequently, has given direction to future interventions strategies aimed at improving the implementation of such recommendations.

## Background

Diabetes Mellitus (DM) has emerged as a major global health concern and the number of DM cases is rising in every country [1]. Over the last decade, China has become the leader in the global DM epidemic [1, 2], and DM prevention and management have become critical public health issues in China [3]. Recent estimates from the International Diabetes Federation (IDF) indicate that in 2015, 109.6 million adults in China had DM and if current trends continue, this figure is projected to rise to 150.7 million by 2040 [1]. Additionally, the World Health Organization have estimated that between 2005–2015, China will forego USD$558 billion due to the premature mortality associated with DM and its related conditions - heart disease and stroke [4].

Type 2 Diabetes Mellitus (T2DM) forms the majority of DM cases (~90–95 %), and is a complex, chronic condition that requires effective long term medical management to prevent or delay chronic complications [5]. A multifaceted range of behavioural, lifestyle and psychological changes are integral to the effective management of T2DM, and patients and health care professionals need to collaborate to ensure clinical and self-care recommendations are adhered to [6]. Traditionally, T2DM management approaches have placed considerable emphasis on blood glucose, cholesterol and blood pressure maintenance within normal range. However, it is now well established that patients with T2DM are at increased risk of developing mental health disorders, and functional problems associated with living with their condition [7]. Indeed, the utilisation of psychological interventions in the management of T2DM has been shown to have direct and measurable benefits for patients, in particular for glycated haemoglobin and psychological status [8–11].

International evidence-based guidelines outline psychological care recommendations for the medical management of patients with T2DM [12–15]. In particular, the Global Guideline for Type 2 Diabetes issued by the IDF recommends the adoption of a patient-centred care approach; the exploration of attitudes, beliefs and worries related to T2DM self-care; the periodic assessment of wellbeing; and the referral to a mental-health care professional when indicated [12]. Additionally, the Chinese Guideline for Diabetes Prevention and Management also recommends psychological care be delivered to support patients in adjusting to their diagnosis and assisting adherence to lifestyle modifications [16]. Despite such recommendations, T2DM management approaches in China are not patient-centred, nor do they recognise the individual as having a central role in the self-management of their condition [17]. Typically, doctors have primarily focused on the provision of medications to manage T2DM, and have largely overlooked the facilitation of behaviour change to moderate or control key T2DM-related outcomes [18, 19]. The observed discrepancy between T2DM clinical recommendations and actual clinical practice in China is likely to be the result of a multitude of organisational and individual factors that influence clinician behaviour. The identification of relevant factors is a necessary step in the development of complex interventions to increase the implementation of evidence-based guidelines [20].

A large pool of psychological theories explaining behaviour change are available to guide implementation research (i.e. theory of planned behaviour [21], social cognitive theory [22]). Many of these theories have overlapping theoretical constructs; there is often no sound basis for selecting among them; and their selection and application for each context frequently requires extensive input from health psychologists. Additionally, the application of just one or a few theories creates the potential for critical theories to be missed. One approach that seeks to make the plethora of theories more accessible and relevant to health service researchers is the Theoretical Domains Framework (TDF). The TDF [23] was developed through an expert consultation process and drew upon 128 theoretical constructs from 33 psychological and organisational theories to produce a single integrative framework that can be used when assessing behavioural difficulties associated with guideline implementation, and when designing interventions. The original TDF has been validated and recently updated to include 14 theoretical domains (comprising 84 component constructs) and is suitable for use in a variety of settings [24] (Table 1). This set of domains has previously been applied by a number of studies in a variety of health care settings including primary care [25], acute care [26], and chiropractic care [27]. Recently, the framework was utilised in Mongolia to explore factors influencing the delivery of hypertension and DM guidelines in primary care settings [28]. Despite the wide utilisation of the TDF framework, no studies to date have been conducted in China and none have investigated the factors related to the implementation of psychological care recommendations in general. As such, this study aimed to examine the barriers and enablers to the implementation of psychological care recommendations for patients with T2DM in China, from the perspective of community health centre (CHC) doctors.

**Table 1:** Domains in the theoretical domains framework

Domain	Definition [24]
1. Knowledge	An awareness of the existence of something
2. Skills	An ability or proficiency acquired through practice
3. Social/professional role & identity	A coherent set of behaviours and displayed personal qualities of an individual in a social or work setting
4. Beliefs about capabilities	Acceptance of the truth, reality, or validity about an ability, talent, or facility that a person can put to constructive use
5. Optimism	The confidence that things will happen for the best or that desired goals will be attained
6. Beliefs about consequences	Acceptance of the truth, reality, or validity about outcomes of a behaviour in a given situation
7. Reinforcement	Increasing the probability of a response by arranging a dependent relationship, or contingency, between the response and a given stimulus
8. Intentions	A conscious decision to perform a behaviour or a resolve to act in a certain way
9. Goals	Mental representations of outcomes or end states that an individual wants to achieve
10. Memory, attention & decision processes	The ability to retain information, focus selectively on aspects of the environment and choose between two or more alternatives
11. Environmental context & resources	Any circumstance of a person’s situation or environment that discourages or encourages the development of skills and abilities, independence, social competence, and adaptive behaviour
12. Social influences	Those interpersonal processes that can cause individuals to change their thoughts, feelings, or behaviours
13. Emotion	A complex reaction pattern, involving experiential, behavioural, and physiological elements, by which the individual attempts to deal with a personally significant matter or event
14. Behavioural regulation	Anything aimed at managing or changing objectively observed or measured actions

## Methods

### Study design

A qualitative study design was employed to determine the barriers and enablers to the implementation of psychological care recommendations for patients with T2DM in China. Specifically, focus group discussions were chosen as the preferred method of data collection due to their interactive nature and their well-established ability to explore people’s knowledge and experiences in relation to a specific behaviour [29]. The study was approved by Monash University Human Research Ethics Committee (Project Number: CF15/1522 - 2015000754).

### Study participants

A convenience sample of 23 CHC doctors from Shenzhen, China were approached face-to-face by a member of the research team (AC) and invited to participate in the present study. Shenzhen is a major city within Guangdong Province, situated in the southeast of China, adjacent to Hong Kong. All CHC doctors were participants in a residential medical training program being conducted in Melbourne, Australia through a joint initiative of the Shenzhen City of Guangdong Province, the Chinese Journal of General Practice and the School of Primary Health Care, Monash University, Australia. Established in 2009, this program aims to enhance the primary health care capacity of leading CHC doctors and intends to build a pool of family medicine ‘champions’ practicing within Shenzhen, China.

### Procedure

All invited CHC doctors (*n* = 23) agreed to participate in the present study. Two focus groups were conducted in May (*n* = 10) and August (*n* = 13) 2015 at Monash University, Melbourne. Written informed consent was obtained from all CHC doctors prior to participation. Focus groups were conducted with a facilitator, and an interpreter fluent in English and Mandarin, and each session ran for approximately 90 min duration. To maintain consistency, both focus groups were conducted by the same facilitator (AC: female, PhD candidate, with previous experience utilising the TDF) and interpreter (HY: male, senior research fellow, who trained as a medical doctor in China). The interpreter was also an educator in the residential training program and was therefore known to all CHC doctors prior to participation in the study. The focus group schedule used to prompt discussion was based on the TDF (version 2) [30] and is outlined in Table 2. Data from focus groups were audio-taped, transcribed verbatim and were entered into NVivo 10 [31] for data management and analysis. To ensure accuracy, both transcripts were reviewed by two authors (AC & HY).

**Table 2:** Focus group schedule and the corresponding theoretical domains

Domain	Focus group schedule
1. Knowledge	• Can you tell me what you know about psychological care in the management of T2DM? • Are you aware of the IDF guideline titled ‘Global Guideline for Type 2 Diabetes - Psychological Care’? - If yes, what is your understanding of the recommendations? - If no [description of the guideline and a copy of the guideline provided to participants]
2. Skills	• How do you usually deliver psychological care to patients with T2DM? - Can you give a specific example of what aspects you deliver? • What skill based training have you received in psychological care?
3. Social/professional role & identity	• What do you think about the relevance of this guideline in the context of CHCs in China? • Do you think it is an appropriate part of your role to be following these recommendations?
4. Beliefs about capabilities	• How difficult or easy is it for you to provide psychological care to patients with T2DM? • What problems have you encountered? - What would help you to overcome these problems?
5. Optimism	• How confident are you that you can deliver the psychological care recommendations despite the difficulties? • What do you think will happen if you do not routinely provide psychological care?
6. Beliefs about consequences	• What do you feel are the consequences of offering psychological care to patients with T2DM (prompt for advantages and disadvantages)?
7. Reinforcement	• How do incentives/rewards influence the delivery of psychological care to patients with T2DM? - What incentives would enhance the delivery of psychological care?
8. Intentions	• Do you intend to deliver psychological care to patients with T2DM? (prompt for further explanation) • Are there other things that interfere with your intentions to deliver psychological care recommendations to patients with T2DM?
9. Goals	• How much do you want to deliver the psychological care recommendations to patients with T2DM? - In what situations would you want to deliver the recommendations?
10. Memory, attention and decision processes	• Do you think to deliver the psychological care to patients with T2DM? • How much attention do you have to pay to deliver psychological care to patients with T2DM? • What are your reasons for not offering psychological care to patients with T2DM? (prompt for attention, forgetting, time constraints, etc.)
11. Environmental context & resources	• To what extent do physical or resource factors facilitate or hinder you in delivering the psychological care recommendations to patients with T2DM? • Are there competing tasks and time constraints that impact the delivery of psychological care recommendations to patients with T2DM? • Do you have the necessary resources available to you to deliver psychological care recommendations to patients with T2DM?
12. Social influences	• To what extent do social influences of peers, CHC staff etc. facilitate or hinder you in delivering psychological care to patients with T2DM? • Do you observe other peers and role models providing psychological care to patients with T2DM? • How do the expectations of your patients and their families influence your provision of psychological care?
13. Emotion	• How do emotional factors influence whether psychological care recommendations are delivered to patients with T2DM? • Does not providing psychological care evoke worry or concern in you?
14. Behavioural regulation	• Are there any procedures or ways of working that encourage or discourage providing psychological care to patients with T2DM? • What do you think is needed to ensure the consistent delivery of psychological care to patients with T2DM?

### Analysis

Data were analysed independently by two of the authors (AC & HY) using deductive thematic analysis [32]. The 14 theoretical domains of the TDF previously described were used as the coding framework (Table 1). Initially, both authors independently classified participant statements into the 14 domains, and all statements could be applied to at least one domain. On completion of coding, both authors compared coding selections and when discrepancies occurred, consensus was reached through discussion with a third author (KS). A summary of participant responses was then tabulated for each of the TDF domains.

## Results

Fourteen women and nine men participated in the study, with all participants employees of urban, government-administered CHCs. The majority of participants held a professional title of attending physician (*n* = 16), with the remainder classified as either associate physician (*n* = 3) or chief physician (*n* = 4). These classifications primarily relate to the experience and skill level of the doctor, which in turn reflects their consultation fee. In China, doctors have a four-level professional title system that is nationally consistent. Increasing in seniority, these titles consist of resident physician, attending physician, associate physician, and chief physician [33]. With regard to highest medical degree obtained, most participants had completed a Bachelor of Medicine (*n* = 17), a further five participants had completed a Masters of Medicine, and one participant a Diploma of Medicine.

Barriers and enablers perceived by CHC doctors as being relevant to the delivery of psychological care for patients with T2DM were primarily categorised within eight of the TDF domains. These were: 1) *knowledge*; 2) *skills*; 3) *social/professional role & identity*; 4) *beliefs about consequences*; 5) *reinforcement*; 6) *memory, attention & decision making*; 7) *environmental context & resources*; and 8) *social influences*. In particular, the TDF domains of *knowledge*; *skills*; *reinforcement;* and *environmental context & resources* dominated the majority of discussion time. The TDF domains that did not emerge from analysis of focus group data included beliefs about capabilities, optimism, goals, intentions, emotion, and behavioural regulation.

Key barriers and enablers, together with illustrative quotes from participants, are presented within the relevant domains below. Text has been placed in brackets when further clarification was deemed necessary for the readability of quotes. Given the potential for the sample to be identified, the characteristics of participants (e.g. age, gender, role, CHC location) have been excluded from quotes to maintain confidentiality of participants. Further, these characteristics (i.e. role, education level) did not appear to influence participant responses. The interrelated domains of knowledge and skills were predominantly referred to simultaneously by CHC doctors, and as a consequence these domains have been combined for presentation.

### Knowledge/Skills

Overall, the principal barrier to the delivery of psychological care to patients with T2DM was the lack of knowledge and skills of CHC doctors in this area. None of the participants were aware of the existence of the psychological care recommendations outlined in the global guideline for T2DM, and when informed of the specific recommendations, all participants felt they did not possess the necessary knowledge and skills to be able to effectively deliver the recommendations. Additionally, the majority of CHC doctors demonstrated a lack of understanding of the term ‘patient-centred care’.

“*Basically, our [CHC] doctors have insufficient knowledge and skills of psychological care. If a patient comes and tells us their worries, their concerns, their anxieties, we basically have no way to respond to their need.” (Participant #3)*

*“We [CHC doctors] previously had a concept of ‘patient-centred care’ in our minds, but we now know that our understanding was totally different.” (Participant #8)*

The medical training received by CHC doctors was commonly cited as a key reason to the lack of knowledge and skills in psychological care. Conversely, the incorporation of a psychological care component into existing medical training and continuing medical education programs were perceived as potential enablers to increase the knowledge and skill base of CHC doctors. Specifically, a need was expressed for practical, skill based training in psychological care that was relevant to their clinical practice.

*“In our medical education, we did not have formal training on mental health and psychological issues, so the current service for mental health is just based on our experience, not from formal education. We need to strengthen the education component to include mental health and psychological skills. We need a ‘toolbox’ to be able to pick up and use for different patients.” (Participant #12)*

### Social/professional role and identity

The majority of CHC doctors felt that the delivery of psychological care to patients with T2DM was a relevant part of their role. However, it was further acknowledged that within the current system, specialist services were largely responsible for the delivery of psychological care in general, with the role of the CHC doctors frequently limited to the detection of severe psychological problems and the monitoring of patients with regard to medication following discharge from a psychiatric hospital.

*“The current situation is that we [CHC doctors] are not recognised as being able to manage common mental health problems. Now, the situation at the system level in China is that we are the informers [referral to specialist hospital following implementation of screening tools] for severe mental disorders.” (Participant #7)*

Whilst perceiving overall that psychological care was relevant to their role when managing patients with T2DM, some CHC doctors did not consider the patient’s level of social support (a component in psychological care recommendations) to be of relevance to their professional role. Additionally, they did not associate social support structures as being influential to a patients’ self-management ability.

*“If a patient came to your clinic and said ‘my girlfriend has left me, please help me’, Chinese doctors would think - Are you crazy? Go away - this is none of my business.” (Participant #15)*

### Beliefs about consequences

Several CHC doctors stated that they were not convinced of the benefits and evidence-base of psychological care for the management of patients with T2DM, and did not believe the delivery of psychological care would offer any additional advantages to their current management approaches.

*“If I deliver psychological care to my patients, will it work? In fact, I doubt that it would because I don’t know the evidence - I don’t know anything. Because of that we [CHC doctors] are not eager to learn psychological aspects [of diabetes care].” (Participant #1)*

Participants also anticipated a negative reaction from their patients if they were to spend time delivering the psychological care recommendations, instead of providing medications to control or manage T2DM-related outcomes.

*“It is difficult. If we provide this service [psychological care], our patients may not think it is important and may not like it. Patients prefer to let us help them to reduce and maintain blood sugar levels - that’s it. That is their wish.” (Participant #19)*

### Reinforcement

This domain includes constructs such as rewards, incentives and punishments that increase the likelihood of a behaviour being performed through the establishment of a dependant relationship with a given stimulus.

The lack of monetary incentives available to CHC doctors for the delivery of psychological care to patients with T2DM was another commonly identified barrier. Participants compared the funding models of the Australian and Chinese primary health care systems and felt that Australian initiatives, such as the Practice Incentives Program (Diabetes Incentive), encouraged the delivery of comprehensive T2DM management. The majority of CHC doctors favoured a restructuring of the current CHC funding model, and believed that for psychological care to be delivered to patients with T2DM in China, a specific incentive would need to be introduced.

*“There are different systems in China and Australia. For example, if you [Australia] complete a care cycle for diabetes patients, the government will pay you. In our system, we are government level doctors and we have a fixed level salary, no matter how many services you provide to the patient. So there is no incentive to provide a service additional to that of the medical service.” (Participant #14)*

### Memory, attention & decision making

Several CHC doctors reported the intentional disregard of the computerised management system that prompts for a psychological assessment to be performed for patients with newly diagnosed T2DM. Only one CHC doctor reported regularly using this feature of the management system.

*“In our management system - the computerised system, we have a template for diabetes management. One of the sections is psychological management. If we find a new case of diabetes, we should perform a mental health status examination. This is based on the computer system, but in reality we do not follow this system.” (Participant #20)*

### Environmental context & resources

Secondary only to the domains of knowledge and skills, time constraints were heavily referred to by CHC doctors as being a major barrier to the delivery of psychological care for patients with T2DM. A high volume of patients (particularly in the morning) commonly resulted in short consultations times, placing limitations on the CHC doctor’s ability to address anything other than the primary reason for CHC attendance.

*“For example, in a 6.5 h day in clinic we will see around 70–80 patients. It is enough for us to handle the medical complaints within this time.” (Participant #16)*

CHC doctors did indicate however, that session times in the afternoon or evening would be more suited to the delivery of psychological care recommendations to patients with T2DM. A number of CHC doctors also stated that they would be more likely to address psychological care if longer consultation times were specifically scheduled. Additional human resources were also favoured, such as the role of a diabetes educator, to assist CHC doctors in delivering psychological care to patients with T2DM.

*“In the morning it would be pretty difficult because many patients come to see me, but in the afternoon or evening we would have some time to provide 30 min to each patient.” (Participant #4)*

*“Maybe we would need to operate a longer consultation or booking to do the [psychological] care - that would be better.” (Participant #3)*

In addition to time, cost was also referred to as a potential barrier for the delivery of psychological care recommendations to patients with T2DM, particularly for patients with a low socio-economic status, and low education level. It was perceived that patients would not be willing to spend more money on a consultation that they didn’t view as essential to their T2DM management.

*“For the lower socio-economic level, and the patients with lower education, they may not worry about the [psychological] complications; they worry about their ability to pay for the service.” (Participant #14)*

### Social influences

The societal perception of psychological health as being less important than physical health was a further identified barrier to the delivery of psychological care. It was also stated that for an increase of psychological care recommendations to occur in the CHC setting, the perception of psychological health in the community would need to shift.

*But everybody in society thinks that mental health is not a big thing, it’s invisible. The only things they [patients] want to see are physical changes, like a blood test after treatment. (Participant #10)*

The lack of guidance from opinion leaders regarding the importance of psychological care for the management of T2DM was also frequently raised by CHC doctors as a source of concern. Opinion leaders such as medical educators, specialists in tertiary hospitals, and the Chinese Diabetes Society were all perceived to have an influential role impacting on the behaviour of CHC doctors.

*“I studied at the Number 1 People’s Hospital - it’s the best hospital in our city, and I was based in the diabetic department. They had diabetes education sessions every week…they educated patients about how to use medicine, also insulin, but mental health care - they never focused on it.” (Participant #18)*

## Discussion

The experiences of CHC doctors indicate that numerous barriers and enablers influence the implementation of psychological care recommendations to patients with T2DM in China. The key barriers perceived by CHC doctors included: knowledge and skill deficiencies in psychological care; time constraints; and the absence of financial incentives. Additional barriers included the societal perception that treating psychological aspects of health is less important than physical health; a lack of opinion leaders; the intentional disregard of psychological care; and doubts regarding the efficacy of psychological care. In contrast, perceived enablers included the provision of psychological skills training to CHC doctors; identification of afternoon and evening clinic sessions as being conducive to implementation of recommendations; introduction of financial incentives; and creation of a professional role that could implement psychological care recommendations to patients with T2DM.

This study has uniquely applied a systematic and theoretical approach to the exploration of behavioural determinants that influence the delivery of psychological care recommendations to patients with T2DM in China. The utilisation of the TDF allowed for the comprehensive understanding of barriers and enablers to the implementation of recommendations, and as such, has given direction to future intervention strategies.

The TDF domains of *knowledge* and *skills* were the most salient factors influencing the non-implementation of psychological care recommendations for the management of patients with T2DM in China. A large body of evidence indicates that firstly, health professionals need to be adequately educated and trained in order to accept and incorporate evidence-based guidelines into their daily practice [34, 35]. Doctors in China do not receive any formal training in psychological care as part of their medical education [36]. As a result, the knowledge and skill base of CHC doctors is generally limited in the counselling and behaviour change techniques that can assist patients in adhering to the complex regimen of T2DM self-care activities [17]. Additionally, the knowledge and skills deficiencies of CHC doctors had a flow-on effect to the domains of *beliefs about consequences* and *memory, attention & decision making.* Specifically, CHC doctors frequently questioned the efficacy of psychological care for the management of T2DM and often opted to intentionally disregard psychological care, even when prompted by their computerised management program. It is likely that the barriers identified in a multitude of TDF domains would be resolved by improving the knowledge and skill base of CHC doctors. As such, interventions should focus on the incorporation of psychological care training into existing medical education and continuing medical education programs. In particular, there is a strong need for practical, skill-based training that is clinically relevant.

The results of the present study also highlight the need to develop strategies to assist time-poor CHC doctors in delivering the psychological care recommendations to patients with T2DM. Consistent with our findings, the majority of implementation research conducted within primary care settings has similarly observed time pressure to be a significant determinant influencing clinician behaviour [25, 28, 37, 38]. The current research has identified a possible opportunity during afternoon and evening clinic sessions for longer consultation times that could potentially be dedicated to the delivery of psychological care to patients with T2DM. To seize this opportunity, interventions should focus on promoting the efficacy of psychological care to patients with T2DM and should advertise the availability of afternoon and evening sessions. This could be achieved via posters in CHC waiting areas; by administrative staff at reception; and by contacting existing T2DM patients.

The establishment of a new professional role, such as a diabetes educator, into existing CHC structures could also potentially relieve the workload pressures faced by CHC doctors. In Hong Kong, the establishment of diabetes nurses within primary care settings has successfully been trialled without compromising the care of T2DM patients [39]. Furthermore, the use of trained nurses as educators has also been found to improve self-efficacy and reduce clinical inertia and non-adherence [40].

Findings in relation to the TDF domain of *reinforcement* indicate that the introduction of financial incentives may enhance the implementation of some aspects of the psychological care recommendations. A synthesis of systematic reviews has previously found financial incentives to increase the implementation of clinical guidelines by up to 39 % [41]. While some elements of the recommendations such as the adoption of a patient-centred care approach cannot be adequately assessed for incentive purposes; the periodic assessment of psychological health has the potential to become incentivised. Recent primary health care reforms in China have resulted in the restructuring to the funding of CHCs. The government now fund the basic salaries of all CHC doctors and additionally provides a per-person subsidy of 15RMB for the delivery of a standard service package that includes preventive care, chronic disease management, primary medical care, rehabilitation, health education, and family planning [42]. It is likely that an extension of the current service package to include the assessment of psychological wellbeing may provide CHC doctors with the adequate motivation to deliver this aspect of the guidelines.

To further enhance the implementation of psychological care recommendations to patients with T2DM, barriers identified within the TDF domain of *social influences* will need to be addressed. Specifically, opinion leaders - such as the Chinese Diabetes Society (the DM branch of the Chinese Medical Association); the Chinese Center for Disease Control and Prevention; medical educators; and specialists within tertiary hospitals are all well placed to promote the implementation of psychological care recommendations to CHC doctors. Additionally, these opinion leaders also have the opportunity to shift the societal perception of psychological health as being inferior to physical health. The Chinese society respects hierarchy and prestige [43] and as such, recommendations and endorsements by these peak bodies are likely to be viewed upon favourably by the public. A potential intervention may therefore be a public health campaign highlighting the need and efficacy of psychological interventions for the management of T2DM that additionally promotes CHCs as the preferred setting to seek this aspect of care.

### Limitations

This qualitative study used a validated framework and adopted a systematic approach in the design and analysis of focus group data. The generalisability of this data is limited because of the fixed number of CHC doctors available for recruitment which contributed to a relatively small sample. Additionally, the sample consisted of leading CHC doctors who were specifically chosen by their CHC to receive specialist training in primary care and may not be representative of all CHCs doctors practicing in Shenzhen, China. The present findings reflect experiences of CHC doctors working within urban settings. The health care system in China differs greatly in both structure and quality between rural and urban settings [44], and it is highly probable that rural doctors in China would generate a different set of barriers and enablers. Further, all CHC doctors in the present study were unaware of the psychological care recommendations for people with T2DM; had the study intentionally sought out participants who were aware of the existence of recommendations, a variation in responses would have been likely.

In addition to the general limitations inherent in all focus group studies [45], cultural characteristics of a sample have been found to influence focus group dynamics and data quality [46]. The Chinese culture is highly collectivistic by nature, whereby individuals subordinate their personal beliefs to the beliefs of a group [43]. Focus groups conducted in collectivistic cultures have previously shown a high level of conformity [46], and this was also the case in the present study, whereby personal characteristics (e.g. professional role, education level) did not appear to influence participant views. As a result, some behavioural determinants may not have been raised by individual doctors for concerns regarding social acceptability. Group dynamics may also have been influenced by the pre-existing relationship between participants and the interpreter (as an educator in the residential training program) which could be considered as having a power differential. Further research, using a combination of quantitative and alternative qualitative methods (i.e. individual interviews) may provide a deeper understanding of the issues raised in this study.

## Conclusion

Using a validated framework, the key barriers and enablers to the implementation of psychological care recommendations for patients with T2DM were identified. This information is a necessary first step in the development of a complex intervention to increase the implementation of recommendations. Indeed, the newly formed International Institute for Primary Health Care Research in Shenzhen, China is leading a methodologically rigorous program of research that intends to increase the implementation and uptake of psychological recommendations for individuals with T2DM. In the short term, future research is planned to determine which of the identified barriers and enablers should be prioritised for intervention. Consideration is also being given to the views of patients with T2DM to the delivery and uptake of psychological care recommendations. Given China’s increasing T2DM burden and the potential for psychological care to reduce the morbidity of patients with T2DM, it is crucial that effective strategies are put in place to deliver the psychological care recommendations.

## Availability of data and materials

The data supporting the conclusions of this article are available upon request from the corresponding author.

## Abbreviations

CHC: Community health centre
DM: Diabetes mellitus
IDF: International diabetes federation
T2DM: Type 2 diabetes mellitus
TDF: Theoretical domains framework
